# PD-1 and PD-L1 expression in pulmonary carcinoid tumors and their association to tumor spread

**DOI:** 10.1530/EC-19-0308

**Published:** 2019-07-12

**Authors:** Tiina Vesterinen, Teijo Kuopio, Maarit Ahtiainen, Aija Knuuttila, Harri Mustonen, Kaisa Salmenkivi, Johanna Arola, Caj Haglund

**Affiliations:** 1 HUSLAB, Department of Pathology, University of Helsinki and Helsinki University Hospital, Helsinki, Finland; 2 Institute for Molecular Medicine Finland (FIMM), Helsinki Institute of Life Science, University of Helsinki, Helsinki, Finland; 3 Department of Biological and Environmental Science, University of Jyväskylä and Department of Pathology, Central Finland Health Care District, Jyväskylä, Finland; 4 Department of Education and Research, Central Finland Central Hospital, Jyväskylä, Finland; 5 Department of Pulmonary Medicine, Heart and Lung Center, and Cancer Center, University of Helsinki and Helsinki University Hospital, Helsinki, Finland; 6 Department of Surgery, University of Helsinki and Helsinki University Hospital, Helsinki, Finland; 7 Translational Cancer Medicine Program, Faculty of Medicine, University of Helsinki, Helsinki, Finland

**Keywords:** neuroendocrine tumor, pulmonary carcinoid tumor, PD-1, PD-L1, immunohistochemistry

## Abstract

Pulmonary carcinoid (PC) tumors are rare tumors that account for approximately 1% of all lung cancers. The primary treatment option is surgery, while there is no standard treatment for metastatic disease. As the number of PCs diagnosed yearly is increasing, there is a need to establish novel therapeutic options. This study aimed to investigate programmed death protein 1 (PD-1) and programmed death ligand 1 (PD-L1) expression in PC tumors since blocking of the PD-1/PD-L1 pathway is a promising therapeutic option in various other malignancies. A total of 168 PC patients treated between 1990 and 2013 were collected from the Finnish biobanks. After re-evaluation of the tumors, 131 (78%) were classified as typical carcinoid (TC) and 37 (22%) as atypical carcinoid (AC) tumors. Primary tumor samples were immunohistochemically labeled for PD-1, PD-L1 and CD8. High PD-1 expression was detected in 16% of the tumors. PD-L1 expression was detected in 7% of TC tumors; all AC tumors were PD-L1 negative. PD-L1 expression was associated with mediastinal lymph-node metastasis at the time of diagnosis (*P* = 0.021) as well as overall metastatic potential of the tumor (*P* = 0.010). Neither PD-1 expression, PD-L1 expression nor CD8^+^ T cell density was associated with survival. In conclusion, PD-1 and PD-L1 were expressed in a small proportion of PC tumors and PD-L1 expression was associated with metastatic disease. Targeting of the PD-1/PD-L1 pathway with immune checkpoint inhibitors may thus offer a treatment option for a subset of PC patients.

## Introduction

Pulmonary carcinoid (PC) tumors are low- and intermediate-grade neoplasms that are subdivided into typical carcinoid (TC) and atypical carcinoid (AC) based on the mitotic count and presence of necrosis (1). PCs belong to pulmonary neuroendocrine tumors together with high-grade large-cell neuroendocrine carcinoma (LCNEC) and small-cell lung cancer (SCLC). However, PCs constitute a totally different entity based on a low number of genetic mutations, low metastatic potential and a generally good prognosis, especially when resected (2, 3, 4). AC tumors tend to have more metastatic potential and thus a more aggressive disease course than TC tumors (2).

The main treatment for local PC tumors is resection while metastatic diseases are treated with cytotoxic agents, radiation therapy and somatostatin analogs, although there is no consensus on these treatments (5). From an epidemiological point of view, PCs are rare tumors accounting for approximately 1% of all lung cancers (6). Still, as the number of PC tumors diagnosed yearly is increasing, mainly due to the increased use of imaging techniques and general awareness of clinicians, there might be a need to establish novel therapeutic options for these patients (7).

Over recent decades, several studies have shown that the immune system plays a critical role in recognizing and eliminating malignant cells. In particular, elevated levels of cytotoxic CD8^+^ T cells in the tumor microenvironment have been linked to a good prognosis, for example in oral squamous cell carcinoma, triple-negative breast cancer and non-small-cell lung cancer (NSCLC) (8, 9, 10). Among other inflammatory cells, CD8^+^ T cells express membrane receptor programmed death protein 1 (PD-1), which interacts with programmed death ligand 1 (PD-L1) expressed for example on the surface of various tumor cells and antigen-presenting cells (11). This interaction of PD-1 and PD-L1 inhibits an antitumor immune response in the tumor microenvironment through downregulation of T cell cytokine production and proliferation (11, 12). Thus, blockading the PD-1/PD-L1 pathway has become a promising therapeutic option for several human malignancies (13). Monoclonal antibodies targeting PD-1 or PD-L1, so called checkpoint inhibitors, are widely used in clinical practice in treating, for example, patients with melanoma or NSCLC (14, 15). In addition, many clinical trials on various cancer types are on-going (13).

Several studies have investigated the expression of PD-1 and PD-L1 in SCLC but reports on PC tumors are rare and have involved only a limited number of patients (16, 17, 18, 19, 20, 21). In addition, in most studies PCs have been merged with high-grade LCNEC and SCLC which behave much more aggressively. As proposed in a recent paper, PCs should be studied independently of LCNEC and SCLC (22). Thus, we collected a large, retrospective, and clinically well-characterized series of surgically resected PC tumors to investigate the PD-1 and PD-L1 expression as well as the number of CD8^+^ intratumoral T cells and their possible relationship to clinicopathologic variables and patient outcome.

## Materials and methods

### Patients

A total of 168 patients operated between 1990 and 2013 were included in the study. Formalin-fixed and paraffin-embedded (FFPE) primary tumor samples and 14 metastatic samples (12 mediastinal lymph-nodes, pleura and tumor tissue adjacent to the superior vena cava) together with patient data were retrieved from Helsinki Biobank (Helsinki, Finland), Auria Biobank (Turku, Finland) and Biobank of Eastern Finland (Kuopio, Finland). Survival data were obtained from the Finnish Population Register Centre and cause of death data from Statistics Finland. Each tumor was re-evaluated from diagnostic whole slides by an experienced pulmonary pathologist following the 2015 classification by the World Health Organization (WHO) of pulmonary neuroendocrine tumors (1). Neuroendocrine differentiation was confirmed by routine immunohistochemical labeling for chromogranin A, synaptophysin and pan-cytokeratin.

One hundred and thirty-one patients (78%) were diagnosed with TC and 37 (22%) with AC tumors. Surgery was the first-line treatment for all patients. Mediastinal lymph-node dissection was performed in 128 patients (76%). Eleven patients (9%) had histologically confirmed lymph-node metastasis at the time of diagnosis, and one of them also had a metastatic lesion in the liver and bones. In addition, 12 patients developed recurrent tumor or distant metastasis in the liver, brain, pleura, mediastinal lymph-nodes or bones during follow-up. Table 1 shows the clinicopathologic features of the patients.

**Table 1:** Clinicopathologic features of the patients.

Variable	TC	AC	All
Sex			
Male	44 (34%)	18 (49%)	62 (37%)
Female	87 (66%)	19 (51%)	106 (63%)
Age			
Mean	53	55	53
Median	55	57	56
Range	19–84	23–77	19–84
Tumor size (cm)			
≤1	34 (26%)	10 (28%)	44 (26%)
1.1–2.9	75 (58%)	19 (53%)	94 (57%)
≥3	21 (16%)	7 (19%)	28 (17%)
Not available	1	1	2
Hilar/mediastinal (N1/N2) nodal involvement at diagnosis			
Yes	5 (5%)	6 (18%)	11 (9%)
No	90 (95%)	27 (82%)	117 (91%)
Not examined	36	4	40
Distant metastasis			
At diagnosis	0	1 (3%)	1 (1%)
During follow-up	5 (4%)	7 (19%)	12 (7%)
Ki-67 labeling index			
<1%	51 (40%)	9 (24%)	60 (36%)
1–2%	64 (50%)	20 (54%)	84 (51%)
>2%	13 (10%)	8 (22%)	21 (13%)
Not available	3		3

AC, atypical carcinoid tumor; TC, typical carcinoid tumor.

As the Finnish Biobank Act provides a lawful basis for research use of patient samples, a project-specific consent was not retrieved. However, this study was approved by the Scientific and Ethical Committees of all three biobanks.

### Next-generation tissue microarray construction

As presented by Zlobec *et al*., next-generation tissue microarray (ngTMA) construction involves careful TMA planning, digital pathology and automated tissue microarraying (23). Briefly, fresh hematoxylin and eosin-stained slides were prepared from each FFPE tissue sample and digitized using a 20× objective. For primary tumors, two representative 1 mm cores from the middle of the tumor as well as two from the tumor border were selected. For metastatic samples, two 1 mm cores were marked. The TMAs were constructed using a TMA Grand Master (3DHISTECH, Budapest, Hungary) or Galileo TMA CK4500 microarrayer (Isenet, Milan, Italy).

### Immunohistochemistry

Fresh 3.5 µm thick TMA sections were cut with a microtome onto positively charged slides. Slides were stained for PD-1 and PD-L1 at the Department of Pathology in the Central Finland Central Hospital and for CD8 at the Department of Pathology in the Helsinki University Hospital as part of a clinical daily routine. After deparaffinization and heat-induced antigen retrieval, sections were incubated with anti-PD-1 (1:50; clone SP269; Spring Bioscience, Pleasanton, CA, USA), anti-PD-L1 (1:100, clone E1L3N, #13684; Cell Signaling Technology) or anti-CD8 (1:50, clone 4B11, NCL-L-CD8-4B11; Novocastra/Leica Biosystems, Nussloch, Germany). Signal visualization for PD-1 and PD-L1 was performed with a Bond Polymer Refine Detection Kit (Leica Biosystems) and for CD8 with ultraView Universal DAB Detection Kit (Ventana Medical Systems, Tucson, AZ, USA). All sections were counterstained with hematoxylin. Fully automated IHC stainer BOND-III (Leica Biosystems) was used for PD-1 and PD-L1 and BenchMark Ultra (Ventana Medical Systems) for CD8. Tonsil served as a positive control for all antibodies.

### Scoring of the staining results

**Figure 1:**
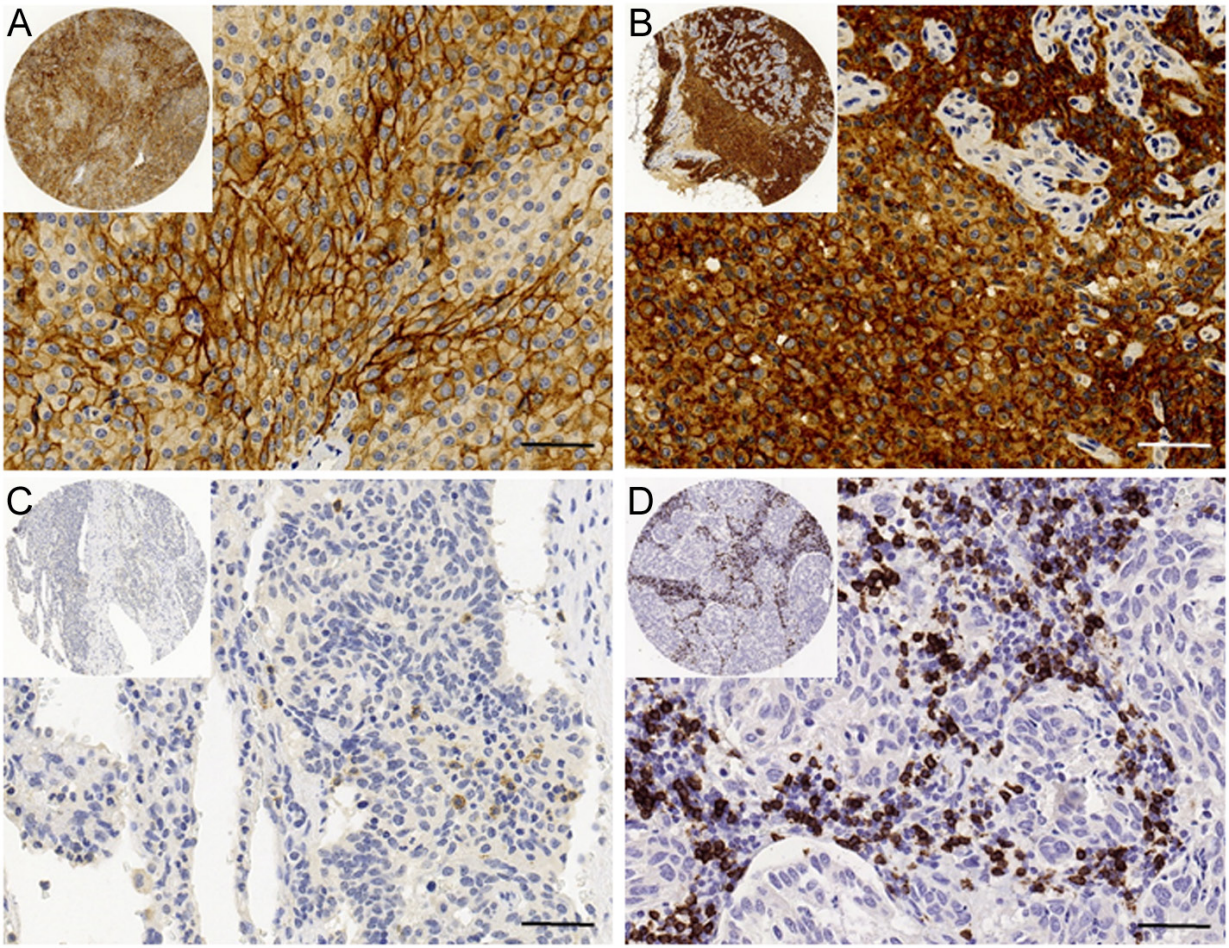
Immunohistochemical images of programmed death ligand 1 (PD-L1), programmed death protein 1 (PD-1) and CD8 staining in pulmonary carcinoid tumors. Positive membranous staining of PD-L1 in a primary typical carcinoid (TC) tumor (A) and in corresponding lymph-node metastasis (B). Intratumoral lymphocytes expressing PD-1 in a TC tumor (C). A TC tumor with an abundant number of intratumoral CD8^+^ T cells (D). Scale bar 50 µm, original magnification 40×. Images obtained from digitized slides with CaseViewer software (3D HISTECH, Budapest, Hungary).

PD-1- and PD-L1-stained slides were digitized with a NanoZoomer-XR scanner (Hamamatsu Photonics, Hamamatsu City, Japan) using a 20× objective. By utilizing NDP.view2 (Hamamatsu Photonics) software for viewing the slides, TK, MA and TV performed PD-1 and PD-L1 scoring manually. No image analysis software was used. A traditional light microscope was used if a digitized image was insufficient. For the assessment of PD-L1, any membranous staining in the tumor cells was evaluated and the proportion of the tumor cell number with PD-L1 staining was scored. As previously described, a score of <1% was considered PD-L1 negative, while a score ≥1% was considered PD-L1 positive (Fig. 1A) (18). The number of PD-1-expressing intratumoral lymphocytes per mm^2^ was manually calculated including both dim and bright expression (Fig. 1C). Expression was categorized into low (≤2 lymphocytes) and into high (>2 lymphocytes) expression based on the median number of PD-1-positive cells (24).

CD8 stained slides were digitized with Pannoramic scanner (3DHISTECH) using a 20× objective. The amount of intratumoral CD8^+^ T cells was assessed with QuPath software by utilizing simple tissue detection and fast cell count functions as previously described by Bankhead *et al*. and Fumet *et al*. (Fig. 1D) (25, 26). Cell counts were measured in each replicate TMA spot and an average value of CD8^+^ T cells per mm^2^ was calculated. Low expression of CD8 was defined as less or equal than the median value (24).

### Statistical analysis

Differences in dichotomous variables between the groups were calculated with the Fisher’s exact test, while the Kruskal–Wallis and the Mann–Whitney *U* tests were used for continuous variables. The Kaplan–Meier method with a log-rank test was used to estimate cumulative survival probabilities. Survival was calculated from the date of surgery to the last date of follow-up or death. A *P* value <0.05 was considered statistically significant. Two-tailed tests were used. Calculations were performed using IBM SPSS Statistics for Windows, Version 24.0 (IBM).

## Results

### PD-1 and PD-L1 expression in PC tumors

PD-1 expression was observed altogether in 68 (40%) tumors (median 2, mean 7, range 1–177 cells per mm^2^). Low expression (≤2 PD-1 positive intratumoral lymphocytes) of PD-1 was found in 41 (24%) tumors and high expression (>2 PD-1 positive intratumoral lymphocytes) in 27 (16%) tumors. High PD-1 expression was associated with younger age (median 51, mean 51, range 20–70 years versus median 60, mean 58, range 29–82 years, *P* = 0.024). PD-1 expression was not associated with histological subtype, gender, size of tumor or Ki-67 labeling index (data not shown).

With the 1% cut-off value, nine tumors (5%) were considered PD-L1 positive (Table 2). All AC tumors were negative. Of ten primary tumor–metastatic sample pairs, two primary tumors were positive for PD-L1. Of the corresponding metastatic samples, one was interpreted as positive and the other as negative (Fig. 1A and B). PD-L1 expression was not associated with histological subtype, gender, age, size of tumor, Ki-67 labeling index or PD-1 expression (data not shown).

**Table 2:** Histological characteristics of typical carcinoid tumors with programmed death ligand 1 (PD-L1) expression.

PD-L1 expression^a^	PD-1 expression^b^	Ki-67 (%)	Tumor size (cm)
95	0	1	1.0
37	0	<1	1.0
14	2	1	0.9
5	0	<1	2.0
4	177	2	3.0
4	3	4	1.3
4	1	2	2.0
2	1	<1	1.5
1	1	9	1.5

^a^% of tumor cells expressing membranous PD-L1 staining; ^b^number of intratumoral PD-1 labeled lymphocytes per mm^2^.

PD-1, programmed death protein 1.

### CD8^+^ T cell density in PC tumors

The median CD8^+^ T cell density was 45 cells per mm^2^ (mean 74, range 2–823) (Fig. 1D). TC tumors showed a slightly lower CD8^+^ T cell density compared with AC tumors (median 45, mean 74, range 2–823 vs median 51, mean 78, range 2–478). However, this difference was not statistically significant (*P* = 0.940). CD8^+^ T cell density was not associated with gender, age, size of tumor or Ki-67 labeling index (data not shown).

### Association of PD-1 and PD-L1 expression with tumor spread

To study the possible relationship between PD-1 expression and metastatic disease, we performed the statistical analysis on all PC patients as well as on TC and AC patients separately. There was no difference in the PD-1 expression in tumors from patients with metastatic disease compared to tumors from patients with non-metastatic disease.

Next, we examined the PD-L1 expression in TC patients with histologically confirmed mediastinal lymph-node metastasis at the time of diagnosis (*n* = 5). Altogether, mediastinal lymph-node dissection was performed in 95 (73%) TC patients, of which five had a PD-L1 positive tumor. We noticed that tumors expressing PD-L1 were accompanied by lymph-node involvements more often than PD-L1 negative tumors (*n* = 89) (2/5, 40% vs 3/89, 3%, *P* = 0.021) were. To further examine the association of PD-L1 expression with tumor spread, we also included the TC patients who developed metastatic disease later (*n* = 4). We observed that tumors expressing PD-L1 were accompanied by lymph-node involvement or distant metastasis more often than PD-L1 negative tumors (3/9, 33 vs 3/88, 3%, *P* = 0.010) were. These data suggest that PD-L1 expression is associated with metastatic potential in TC tumors.

### Association of CD8^+^ T cell density with tumor spread

Furthermore, we studied the possible association of CD8^+^ T cell density with metastatic disease. The median CD8^+^ T cell density was higher in tumors that had metastasized into mediastinal lymph-nodes at the time of diagnosis compared to non-metastatic tumors (65 vs 43, range 21–227 and 2–823 cells per mm^2^, respectively). However, this difference was not statistically significant (*P* = 0.162). The same was true when including patients, who developed distant metastases during the follow-up (*n* = 8): median CD8^+^ T cell density was 59 for these tumors and 44 for tumors without metastasis (*P* = 0.375). We either found no difference in CD8^+^ T cell density between metastatic and non-metastatic tumors when evaluating TC and AC patients separately.

### Association of PD-1, PD-L1 and CD8^+^ T cell density with survival

The median time for patient follow-up was 11.5 years (average 12.7 years, range 1.1–28.0 years). During the follow-up, 4 TC patients and 5 AC patients died from disease, and 23 patients died from unrelated causes. None of the patients whose tumor was expressing PD-L1 died from disease. Neither PD-1 or PD-L1 expression nor CD8^+^ T cell density was associated with disease-specific mortality (data not shown).

## Discussion

PCs are uncommon neoplasms with a rising incidence and prevalence. This rise together with limited therapeutic options for metastatic disease has accentuated the need for new treatment strategies. In this regard, the immune checkpoint-based therapy targeting PD-1 or PD-L1, that has shown its efficacy in other malignancies, could be a feasible option for the treatment of metastatic or inoperable PC tumors. In the present study, involving the so far largest PC tumor cohort published, we evaluated immunohistochemically the PD-1 and PD-L1 expression together with the intratumoral CD8^+^ T cell density in a set of clinically well-characterized, resected PC tumors.

First, we noticed that PCs, especially TCs, can express PD-L1. To the best of our knowledge, only four studies on PD-L1 expression in PCs have been published earlier (16, 18, 20, 21). In all studies, PCs were pooled with LCNEC and SCLC, and patient numbers were relatively low, ranging from 22 to 57. In particular, AC patients were under-represented (2, 6, 11 and 18 patients). Three studies used whole tissue sections for PD-L1 analysis and one utilized TMAs. All studies used different primary antibodies (SP142 (ZSGB-BIO); E1L3N (Cell Signaling); ab205921 (Abcam) and 22C3 (Dako)). In contrast to our results, two of the studies observed no PD-L1 expression in PCs even if the same primary antibody was used as we did (18, 20). In line with our results, Wang *et al*. found PD-L1 expression in 9% of the TC tumors, while all AC tumors were PD-L1 negative (21). Fan *et al*. did not differentiate between TC and AC but reported that 59% of the PCs expressed PD-L1 (16). However, they also interpreted cytoplasmic staining, while we focused on evaluating only membranous staining. The PD-1 expression in PC tumors is less studied but Fan *et al*. reported a 59% positivity for PD-1 in their PC tumors and an association between PD-1 expression and worse prognosis (16). We found PD-1 positivity in 16% of the tumors with no association to prognosis.

Secondly, we observed that the PD-L1 expression in TC tumors was associated with metastatic disease. Previous studies on PCs found no association between PD-L1 expression and lymph-node involvement or distant metastasis (16, 21). However, neither of the studies analyzed PCs separately but merged them with LCNEC and SCLC.

We found no association between the PD-L1 expression and survival, probably due to the low number of disease-specific deaths. In contrast, Wang *et al*. reported that the expression of PD-L1 in tumor cells showed a trend toward poorer overall survival and progression-free survival, although statistical significance was not reached in their study either (21). Fan *et al*. reported PD-L1 expression in cancer cells to be an independent favorable prognostic factor (16).

As discussed, there are controversial reports on the PD-1 and PD-L1 expression and their possible relations to tumor spread and disease outcome in PC tumors. However, studies indicate that there is a subset of patients whose tumors express PD-1 or PD-L1 and that the expression associates with patient outcome. Unfortunately, due to the lack of standardization of the immunohistochemical methods especially in PD-L1 analysis, there is a lot of variation in the primary antibodies, staining instruments and detection protocols as well as scoring criteria applied. The variation partly explains discordant results.

Cancer immunotherapy with monoclonal antibodies is a promising treatment especially for melanoma, NSCLC and Hodgkin’s lymphoma (27, 28, 29). The U.S. Food and Drug Administration has approved two checkpoint inhibitors targeting PD-1 (pembrolizumab and nivolumab) as well as three PD-L1 targeting agents (atezolizumab, avelumab and durvalumab) (30). Of these, pembrolizumab has also been studied in patients with neuroendocrine tumor. The KEYNOTE-028 study (NCT02054806) enrolled patients with PD-L1 positive, well- or moderately differentiated pancreatic neuroendocrine tumor (*n* = 16) or carcinoid tumor (*n* = 25 of which nine were PCs) (31). It showed that pembrolizumab is generally well tolerated and that 10% of the patients had an objective response. The stable disease rate was 71%. The efficacy and safety of pembrolizumab was also studied in the KEYNOTE-158 study (NCT02628067) of patients with well- and moderately differentiated neuroendocrine tumor of the lung, appendix, small intestine, colon, rectum or pancreas (*n* = 107) (32). In this study, pembrolizumab showed antitumor activity with an overall response rate of 3.7% (4/107 patients, 95% confidence interval 1.0–9.3), all responders having a PD-L1-negative tumor. Sixty-one (57%) patients showed stable disease as the best response. Both studies show that anti-PD-1 monotherapy is beneficial for a subset of NET patients. However, as only a limited number of patients benefited from the treatment, for example combination therapies are needed to potentiate the efficacy of immune checkpoint inhibitors. At the moment, PD-L1 expression is the best known biomarker for selecting the patients for anti-PD1/PD-L1 therapy but for NET patients, it does not seem to be an optimal one. Thus, also new predictive biomarkers are urgently needed.

There are a couple of limitations in our study. Firstly, the number of disease-specific deaths is low, despite the relatively long follow-up time, resulting in the fact that we could not properly estimate the relation between PD-1, PD-L1, CD8^+^ T cell density and survival. Secondly, we used TMAs instead of whole sections and might thus have missed some PD-1-or PD-L1-positive areas or hot spots for CD8^+^ T cells despite comprehensive ngTMA approach. However, recently Elfving *et al*. showed that the proportion of PD-L1 expression is comparable between resection specimens and TMA sections (33). They observed that 9% of the TMA cases showed different PD-L1 status when compared to the resection specimen. In our opinion, the use of TMA slides does not influence scientific conclusions in studies on large patient series like ours. Thirdly, due to the retrospective nature of the study dating back to the 1990s, lymph-node dissection was not performed in all patients and thus we might have misclassified some locally advanced diseases. On the other hand, our study comprises a large number of clinically well-characterized patients. We also appropriately re-evaluated each tumor according to the latest WHO classification and utilized clinically validated protocols for immunohistochemical labeling of PD-1, PD-L1, and CD8.

In conclusion, our study shows that some PCs, especially TCs, express PD-L1, which is associated with lymph-node involvement at the time of diagnosis as well as overall metastatic potential of the tumor. High PD-1 expression was found in 16% of the tumors. As only a minority of the PC tumors expressed PD-L1 and there was only a limited number of intratumoral T cells positive for PD-1, monotherapy with PD-1/PD-L1 inhibitors might benefit a subset of PC patients.

## Declaration of interest

The authors declare that there is no conflict of interest that could be perceived as prejudicing the impartiality of the research reported.

## Funding

This work was supported by the Finnish Cancer Foundation (no grant number) and the Helsinki University Hospital Research Fund (grant number TYH2017204).

## Author contribution statement

Tiina Vesterinen: conceptualization, formal analysis, project administration, investigation, writing – original draft, visualization. Teijo Kuopio: investigation, formal analysis, writing – review and editing. Maarit Ahtiainen: investigation, formal analysis, writing – review and editing. Aija Knuuttila: investigation, resources, writing – review and editing. Harri Mustonen: formal analysis, writing – review and editing. Kaisa Salmenkivi: investigation, formal analysis, writing – review and editing. Caj Haglund: resources, funding acquisition, writing – review and editing. Johanna Arola: conceptualization, resources, supervision, funding acquisition, writing – review and editing. J Arola and C Haglund: equal last authorship.
